# Differentiation of Polyamide 6, 6.6, and 12 Contaminations in Polyolefin-Recyclates Using HPLC Coupled to Drift-Tube Ion-Mobility Quadrupole Time-of-Flight Mass Spectrometry

**DOI:** 10.3390/polym13122032

**Published:** 2021-06-21

**Authors:** Andrea Schweighuber, Jörg Fischer, Wolfgang Buchberger

**Affiliations:** 1 Institute of Analytical Chemistry, Johannes Kepler University, Altenbergerstraße 69, 4040 Linz, Austria; wolfgang.buchberger@jku.at; 2 Institute of Polymeric Materials and Testing, Johannes Kepler University, Altenbergerstraße 69, 4040 Linz, Austria; joerg.fischer@jku.at

**Keywords:** recyclates, polyamide, contaminations, liquid chromatography, ion mobility

## Abstract

Recycling is a current hot topic with a focus especially on plastics. The quality of such plastic recyclates is of utmost importance for further processing because impurities lead to a reduction thereof. Contaminations originating from other polymers are highly problematic due to their immiscibility with the recyclate, leading to possible product failures. Therefore, methods for the determination of polymer impurities in recyclates should be investigated. In this paper, an approach for the identification of three different polyamide grades (polyamide 6, 6.6, and 12) is presented, applicable for the analysis of polyolefin-recyclates. An HPLC equipped with a drift-tube ion-mobility QTOF-MS was used for the identification and differentiation of compounds originating from the polyamides, which were then used as markers. These marker compounds are specific for each type and can be identified by their corresponding value of the collision cross section (CCS). After a simple sample preparation, all three types of polyamides were identified within one measurement. In particular, the problematic differentiation of polyamide 6 and 6.6 was easily made possible.

## 1. Introduction

Worldwide major objectives are required to be set in order to manage the increasing amount of waste generated every day. In the European Union (EU) alone, 2327 million tons of waste were generated by the industry and households in 2018 [1]. These huge amounts need to be reasonably reused in order to conserve scarce resources. A focus was set on the implementation of a circular economy by life cycle assessments and waste management strategies. The EU positioned itself as a pioneer by enforcing programs and directives, such as the Environment Action Programme, having the main goals of waste reduction, recycling, and limitation of incineration and landfill of waste [2]. A differentiation of these wastes and waste streams is necessary, due to the different encountered challenges. Recently, a focus on plastic waste and the handling thereof was initiated. The Directive (EU) 2018/852 specifically targets minimum recycling quotas for plastic waste of 50% by 2025 and of 55% by 2030, respectively [3].

Different sources of plastic waste can facilitate or complicate further processing. Waste streams originating from the primary production of plastics can easily be recycled due to the purity of the polymer. Plastics deriving from municipal waste possess a more complex and heterogeneous composition, and hence a crucial component of mechanical recycling is proper sorting to generate pure streams. Solely homogeneous recyclates are suitable for the production of valuable products and for representing a real alternative to virgin materials [4,5]. In 2018, 29.1 Mt of post-consumer plastic waste was collected in the EU, whereby 32.5% was recycled (42.6% was used for energy recovery and 24.9% was landfilled). Approximately 17.8 Mt of this waste consisted of post-consumer packaging waste, reaching a recycling rate of 42% (39.5% was used for energy recovery and 18.5% was landfilled).

The most prominent polymers in packaging applications are polyolefins, namely high- and low-density polyethylene and polypropylene [6]. Polymer contaminations in polyolefin-recyclates can be problematic due to the immiscibility of different polymer mixtures, and these inhomogeneities can lead to a reduction of mechanical properties and thus to eventual product failures [4,7,8,9,10]. Therefore, it is important to acquire a general understanding of the composition of municipal plastic wastes through the analysis of the origin of polymer contaminations [11,12]. Such impurities often derive from composite materials consisting of more than one polymer type, for example, multilayer films used in the food packaging industries. [13,14] Polyamides (PA) are used excessively: 41% of simple multilayer films consist of polyolefin/polyamide blends and a share of 30% is reached with regard to thermoformed packaging films [15].

Polyamides have several advantages dependent on the type of PA. Polyamide 6, polymerized using ɛ-caprolactam, offers great long-term heat resistance, polyamide 6.6, produced from 1,6-hexamethylenediamine and adipic acid, has excellent gas barrier properties, and polyamide 12, synthesized with aminolauric acid or dodecalactam, possesses the lowest water absorption of all polyamide grades [16,17,18].

PA is one of the major polymer contaminants in polyolefin-recyclates and methods for the analysis of low amounts of PA should be developed in order to provide essential information on recyclate quality. Generally, quality control measurements of recyclates are often performed using differential scanning calorimetry (DSC), thermogravimetric analysis (TGA), melt-flow index (MFI), or Fourier-transform infrared (FTIR)-spectroscopy. DSC and TGA can first be approached for a statement about the purity of the polymer, yet a clear differentiation of the contamination is difficult, especially with regard to different types of polyamides [5,19]. Pure polyamides are often characterized by IR spectroscopy, yet due to the low concentration in polyolefin-recyclates, interfering signals of additives containing amide groups complicate this analysis technique. Similarly, another technique applicable is DSC, whereby polyamides can be distinguished, but again, low concentrations pose a problem [20,21]. For waste materials containing high amounts of polyamides (>60%), Zagar et al. developed a method for the analysis of PA 6 and 6.6 by a total hydrolysis of the polymer to small linear species, followed by an HPLC-MS method for the quantification [22]. However, this method is not applicable for the determination of PA contaminations in polyolefin-recyclates because of possible incomplete hydrolysis of the polyamides due to the polyolefin matrix and the low amount of polyamide present in these recyclates.

Therefore, alternative methods for the analysis of low contents of polyamides need to be established for recyclates. An attractive new strategy can be based on targeting not the polymer itself, but low-molecular-mass linear or cyclic oligomers existing in PA and serving as markers for the presence of PA in recyclate materials. Methods for the analysis of such oligomers already exist in the context of risk assessments of PA food contact materials using migration experiments [23,24,25,26,27,28,29,30]. To date, this approach has also been applied to polyolefin-recyclates containing polyamide 6, and included an extraction of the cyclic oligomers followed by HPLC-MS analysis [31]. Yet, to evaluate which types of polyamides are present and to later generate a sum parameter of total polyamide contaminations, this method has limitations. In particular, the differentiation of polyamides with the focus on the three predominant grades PA 6, PA 6.6, and PA 12 has not yet been achieved. Dependent on the type of polyamide, different analytes suitable as marker substances should be further investigated. A predominant problem is the differentiation of these markers due to their similarities in structure, exact mass, and retention time. Especially if a combination of polyamide grades is present in a recyclate, a differentiation by HPLC-MS is not possible. Therefore, an additional level of separation needs to be introduced by usage of a drift-tube ion-mobility mass spectrometer. Through the different shapes of the molecules, differences in the spherical size in the gas phase exist. This fact can then be used in the drift-tube to separate molecules even when they have the same exact mass and retention time. The resulting collision cross section is a unique parameter for each marker compound, which can be calculated by the individual drift times of the molecules.

Therefore, the aim of this work is the differentiation of polyamides using an HPLC coupled to a drift-tube ion-mobility quadrupole/time-of-flight mass spectrometer (DT-IM-QTOF-MS), later applicable for polyolefin recyclates. This research helps to develop a better understanding of the origin of polyamide contaminations in different waste streams, leading to possible improvements in recycling by reduction of these impurities.

## 2. Materials and Methods

### 2.1. Chemicals

All chemicals used were of analytical grade. The extraction solvents methanol and toluene were purchased from VWR International GmbH (Darmstadt, Germany). High-purity water (18 MΩ cm) generated from a Millipore purification system (Molsheim, France), acetonitrile and formic acid (>99%) from VWR International GmbH (Darmstadt, Germany), and ammonium formate (97%) purchased from Sigma Aldrich Handels GmbH (Vienna, Austria) were used for the mobile phases. The analyzed polyamide 6, 6.6 and 12 samples were commercially available from local manufacturers.

### 2.2. Instrumentation

Method development and qualitative analysis were performed on an Agilent 1260 HPLC system, comprising a 1260 flexible pump, a 1290 MCT, and a 1260 autosampler (Agilent Technologies, Santa Clara, CA, USA) coupled to a 6560 DTIM QTOF-MS equipped with a Dual Agilent Jet Stream Electrospray Ionization (Dual AJS-ESI) source and a gas kit (Alternate Gas Kit, Agilent Technologies, Santa Clara, CA, USA). A gradient elution was performed using an XBridge BEH Amide HILIC column (2.1 mm × 100 mm, 2.5 µm; Waters, Milford, MA, USA) equipped with an XBridge Glycan BEH Amide guard column (2.1 mm × 5 mm, 2.5 µm; Waters, Milford, MA, USA). A mobile phase flow of 0.3 mL min^−1^ was employed and the column was maintained at 30 °C. Injection volume was 2 µL.

The optimized mobile phase consisted of 5 mM ammonium formate in water containing 0.1% formic acid (A), acetonitrile with 0.1% formic acid (B), and 100 mM ammonium formate in water containing 0.1% formic acid (C). The gradient elution was performed as follows: starting with 15% A and 85% B, held for two minutes, followed by a linear increase to 15% A, 55% B, and 30% C from minutes 2 to 8, and held constant for 4 min. The gradient was changed to the starting conditions within 0.5 min and held for 7.5 min for re-equilibrating the column.

The ESI source was operated in the positive mode. As sheath and drying gas, nitrogen, was used. Sheath gas temperature was set to 350 °C at a flow rate of 11 L min^−1^. The temperature of the drying gas was 250 °C using a flow of 10 L min^−1^. The nebulizer pressure was set to 35 psi, the fragmentor voltage to 400 V, the capillary voltage to 3500 V, and the nozzle voltage to 1000 V. An auto-tune in the 2-GHz extended dynamic range setting in the 1700 m/z mode was performed. A 4-bit pulsed multiplexing was applied, using a trap fill time of 3900 µs and a trap release time of 250 µs. The frame rate was set to 0.9 frames s^−1^, the IM transient rate was 18 transients frame^−1^ and the maximum drift time was 60 ms. For the calculations of the collision cross section (^DT^CCS_N2_) using a drift-tube (DT) and nitrogen as collision gas, a single field calibration was performed using an Agilent tuning mix. The applied IM parameters for the DT measurements were in accordance with [32]: 1574 V drift-tube entrance voltage, 224 V drift-tube exit voltage, 217.5 V rear funnel entrance voltage and 45 V rear funnel exit voltage.

### 2.3. Sample Preparation

A microwave-assisted extraction was performed using 100 mg sample and 2 mL solvent (methanol/toluene, 50/50) in G4 reaction vessels (Anton Paar GmbH, Graz, Austria). The microwave was a Monowave Extra equipped with a MAS24 autosampler (Anton Paar GmbH, Graz, Austria). The following parameters for the extraction were applied: a constant temperature of 160 °C was held for 20 min with stirring at a speed of 600 rpm, followed by a cooling step to 40 °C. The samples were then further filtrated in HPLC glass vials using Chromafil AO-45/25 RC filters (Macherey Nagel, Düren, Germany). A solvent change to acetonitrile was done by the evaporation of 500 µL of each sample to dryness under a nitrogen stream and reconstitution with 500 µL acetonitrile. Prior to the analysis, the samples were diluted by a factor of 10 if the concentration exceeded 10 wt% of polyamide content in a recyclate.

### 2.4. Data Evaluation

The Agilent MassHunter LCMS Acquisition software 10.0 was used for the data acquisition. The multiplexed data were then further processed with the PNNL PreProcessor 3.0 software for de-multiplexing the data. IM data was first calibrated with the single field tune using the IM-MS Browser software. The determination of the ^DT^CCS_N2_ values was conducted using the feature extraction (IMFE) in the IM-MS Browser with the following settings: Chromatographic processing of “common organic molecules” with a limited charge state of z ≤ 2. The ion intensity was set to ≥100 and the retention time was restricted to 1–4 min.

The software Agilent MassHunter Qualitative Analysis B.10.00 and Agilent IM-MS Browser 10.0 were employed for data evaluation.

## 3. Results and Discussion

**Figure 1:**
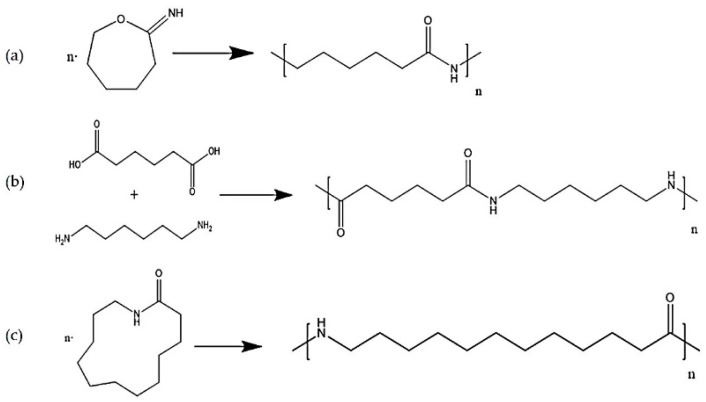
Reaction equations for the polymerization of polyamide 6 (**a**), polyamide 6.6 (**b**), and polyamide 12 (**c**).

The first step towards a qualitative method for the analysis of polyamides was the identification of relevant marker molecules, which are representative for the different types of polyamides. Therefore, the samples were prepared by using an optimized extraction procedure and analyzed by HPLC-QTOF-MS using a HILIC column as was previously done for PA 6 [31], where cyclic compounds related to ɛ-caprolactam were identified. As shown in Figure 1, similar cyclic compounds can be expected for PA 6.6 and PA 12 due to their similar chemical structures by the polymerization reaction for these three types of PA. Polyamide 6 and 6.6 only vary in the location of the functional groups. Polyamide 6 is polymerized using ɛ-caprolactam (a), polyamide 6.6 is produced from 1,6-hexamethylenediamine and adipic acid (b), and polyamide 12 is synthesized with aminolauric acid or dodecalactam (c).

For the three polyamide types, relevant marker compounds were identified, as summarized in Table 1. Structures are given in Tables S1–S3 in the electronic supplementary materials. Chromatographic separation of marker molecules of PA 6 and PA 6.6 were only achieved by using a HILIC column. Several problems occurred using an RP column such as co-eluting peaks and peak tailing. Different HILIC columns were tested, using either diol, ethylene bridged hybrid (BEH) amide, or zwitterionic materials as packaging. Best results were obtained using the BEH amide HILIC column. Throughout all measurements, default values of the ESI ion source were used due to sufficient signal intensities of the marker molecules.

These marker compounds can be used for the identification of the different polyamide grades if these are individually present in the recyclate. In case of mixtures of PAs, the cyclic trimer and cyclic pentamer are selective markers for PA 6. Therefore, the presence or absence of the corresponding peaks in the chromatogram are an unequivocal evidence for the presence or absence of PA 6 in a recyclate. However, for a mixture of PA 6 and PA 6.6 the proof for the presence of PA 6.6 becomes much less straightforward. The marker compounds identified for PA 6.6 are isomers of the cyclic dimer/tetramer/hexamer of PA 6 (see Table 2), so that a chromatographic separation is difficult and a mass spectrometric differentiation is hampered due to the same exact masses. A way around this problem may be the use of the ratio between the total peak area of the two coeluting tetramers (resulting from both PA 6 and PA 6.6) and the peak area of the trimer (resulting from PA 6). However, this approach becomes unreliable when one PA is present in a large excess over the other one.

**Figure 2:**
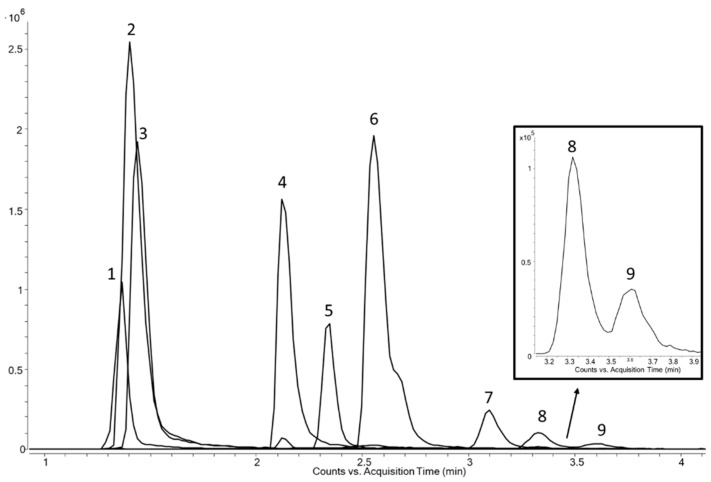
Extracted ion chromatogram of PA 6, PA 6.6, and PA 12. Peaks: 1 = cyclic trimer of PA 12; 2 = cyclic dimer of PA 12; 3 = cyclic monomer of PA 12; 4 = cyclic dimers of PA 6/6.6; 5 = cyclic trimer of PA 6; 6 = cyclic tetramers of PA 6/6.6; 7 = cyclic pentamer of PA 6; 8 = cyclic hexamer of PA 6.6; and 9 = cyclic hexamer of PA 6.

A thorough optimization of the chromatographic separation was done by an adaption of the gradient elution using a higher amount of salt, leading to the final conditions mentioned in Section 2.2. A chromatogram of a mixture of PA 6, PA 6.6, and PA 12 can be seen in Figure 2. The cyclic compounds related to PA 12 (peaks 1–3) elute shortly after the injection peak and cannot be separated by a HILIC column. The cyclic dimer of PA 6 and PA 6.6 coelute (peak 4), and their cyclic tetramers lead to a peak with a shoulder (peak 6). The best that could be achieved was a partial peak separation of the two cyclic hexamers of PA 6 and PA 6.6 with a calculated resolution close to 1 (peaks 8 and 9). However, even this resolution may be too poor when the two compounds are present at quite different levels. Thus, the two often used identification features, exact mass and retention time, are not fully feasible for distinguishing the polyamide grades 6 and 6.6.

**Figure 3:**
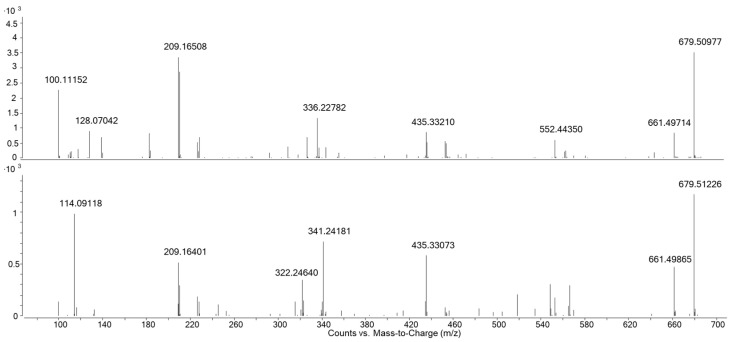
MS/MS data of the cyclic hexamer of PA 6.6 (**top**) and PA 6 (**bottom**), recorded with a collision energy of 40 V.

Another possibility for the differentiation of compounds are mass spectrometric fragmentation patterns and the use of characteristic fragmentation products. A comparison of the MS/MS spectra of the cyclic hexamers of polyamide 6 and 6.6, recorded with a collision energy of 40 V can be seen in Figure 3. Most signals are identical; the main difference which can be found is the fragment with a m/z of 114.09 only visible in PA 6, which corresponds to ɛ-caprolactam. However, there is no specific fragment for PA 6.6 and therefore a differentiation of these two polyamide grades is not possible.

Therefore, an alternative unique parameter is desirable for the clear identification of these marker compounds. This can be accomplished by introducing a further dimension of separation through the use of ion mobility in the mass spectrometer, whereby the ionized molecules are separated in the gas phase according to their shape and charge. Bigger molecules travel longer through the drift-tube, caused by more collisions with the drift gas compared with smaller ones. The intention of using ion mobility was the idea that the cyclic isomeric compounds are folded differently, dependent on the location of the functional groups. The results of the IM-measurements are presented in Table 3.

It can be seen that the retention times of the marker compounds of PA 12 are rather low, due to their low polarity and they elute simultaneously. However, through the collision cross section, a clear parameter is given for an easy differentiation. In the case of PA 6 and PA 6.6, all compounds with the identical mass have the same retention time (except for the cyclic hexamer), yet drift times and therefore the ^DT^CCS_N2_ are different. The difference of the CCS value increases with increasing mass of the oligomer, indicating that the idea of different folding being dependent on the position of the functional groups is valid. Limitations of this approach can be seen with the smaller molecules. Here, the resolution of the drift-tube is not sufficient for a peak separation in the drift spectra. Future advances in the drift-tube ion-mobility technology can be expected to lead to further improvements in this field. Another possibility to enhance resolution is the use of recently developed, sophisticated software packages for high-resolution demultiplexing which significantly increase the resolution power [32] but have not yet been available for this work.

**Figure 4:**
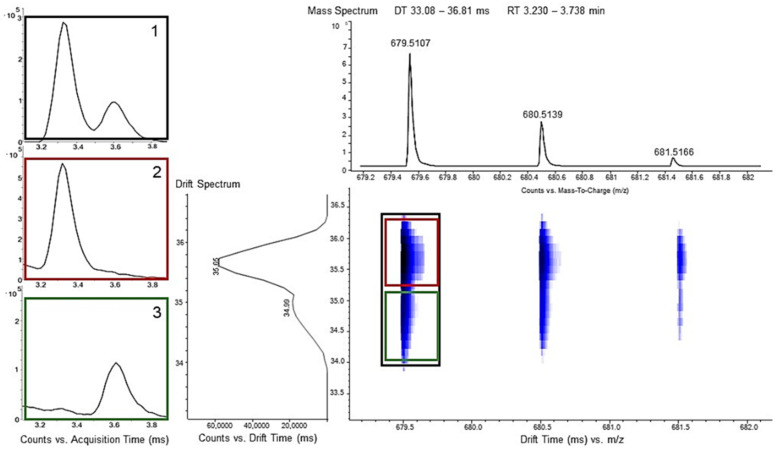
MS and DTIM data for the cyclic hexamer of a mixture of PA 6 and 6.6. At the exact mass of 679.511, a double peak in the drift spectrum can be observed, originating from the two different marker molecules, which vary only in the positioning of the functional groups. Extracted ion chromatogram of 1: drift time window of 33.1–36.8 ms, 2: drift time window of 35.0–36.8 ms, and 3: drift time window of 33.08–35.0 ms.

Best suited for the qualitative differentiation of PA 6 and PA 6.6 is the cyclic hexamer, because a separation by chromatography is partly possible and the additional CCS value guarantees a clear distinction, as depicted in Figure 4.

It can be seen that when measuring a mixture of PA 6 and PA 6.6, the drift sprectrum shows at the exact mass of the cyclic hexamer (m/z: 679.5107) not a single peak but a split peak. This means that at this specific mass not only is one ionized molecule species present but two, which possess two different drift times. This can be used to extract a specific drift time to differentiate the two peaks in the chromatogram.

If the chromatogram is extracted over the whole drift time of 33.1–36.8 ms, the double peak of the cyclic hexamer is observed. However, if only one of the drift times, either from 35.0–36.8 ms or from 33.08–35.0 ms is selected, one specific hexamer is extracted selectively. At the higher drift time, the hexamer of PA 6.6 is visible and at the lower one, the hexamer of PA 6. Therefore, dependent on the drift time, or the resulting CCS-value, polyamide 6 and 6.6 can be distinguished. This qualitative approach is feasible down to a concentration of 0.2 wt% polyamide contamination in polyolefin recyclates.

## 4. Conclusions

In this study, a method for the qualitative differentiation of polyamide 6, 6.6, and 12 was presented by the usage of ion-mobility as an additional separation step. All found marker compounds were characterized by means of determination of the exact mass, fragmentation pattern, retention time, and CCS value. Therefore, it can be guaranteed that these compounds can be differentiated from one another and hence are applicable for the differentiation of polyamide grades. This method is not only applicable if one of these polyamides is present but as well for mixtures. By using ion-mobility, a differentiation of polyamide 6 and 6.6 was achieved by comparison of the resulting CCS values.

## Figures and Tables

**Table 1:** Summary of identified marker compounds for the three polyamide grades.

Polyamide Type	Name	Chemical Formula	Theoretical m/z	Experimental m/z	Retention Time/min
PA 6	ε-caprolactam, Azepan-2-one	C_6_H_11_NO	114.0919	114.0907	1.723
	caprolactam cyclic dimer, 1,8-Diazacyclotetra-decane-2,9-dione	C_12_H_22_N_2_O_2_	227.1760	227.1773	2.119
	caprolactam cyclic trimer, 1,8,15-Triazacyclo-heneicosane-2,9,16-trione	C_18_H_33_N_3_O_3_	340.2600	340.2591	2.326
	caprolactam cyclic tetramer, 1,8,15,22-Tetraazacyclo-octacosane-2,9,16,23-tetrone	C_24_H_44_N_4_O_4_	453.3441	453.3452	2.552
	caprolactam cyclic pentamer, 1,8,15,22,29-Pentaazacyclopenta-triacontane-2,9,16,23,30-pentone	C_30_H_55_N_5_O_5_	566.3890	566.4271	3.098
	caprolactam cyclic hexamer, 1,8,15,22,29,36- Hexaazacyclodotetracontane-2,9,16,23,30,37-hexone	C_36_H_66_N_6_O_6_	679.4339	679.5105	3.606
PA 6.6	1,7-Diazacyclotetra-decane-8,14-dione	C_12_H_22_N_2_O_2_	227.1760	227.1770	2.119
	1,7,15,21-Tetraazacyclo-octacosane-8,14,22,28-tetrone	C_24_H_44_N_4_O_4_	453.3441	453.3449	2.683
	1,7,15,21,29,35- Hexaazacyclodotetracontane-8,14,22,28,36,42-hexone	C_36_H_66_N_6_O_6_	679.4339	679.5112	3.324
PA 12	laurolactam, Azacyclcotridecane-2-one	C_12_H_23_NO	198.1858	198.1860	1.440
	1,14-diazacyclohexacosane-2,15-dione	C_24_H_46_N_2_O_2_	395.3638	395.3635	1.403
	1,14,27-triazacyclononatriacontane-2,15,28-trione	C_36_H_69_N_3_O_3_	592.5417	592.5404	1.365

**Table 2:** Structures of the identified cyclic marker compounds for PA 6 and PA 6.6 having the identical exact mass.

	PA 6	PA 6.6
cyclic dimer theoretical m/z: 227.176	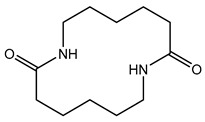	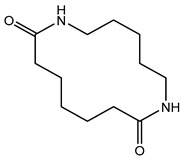
cyclic tetramer theoretical m/z: 453.344	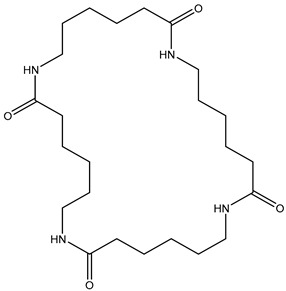	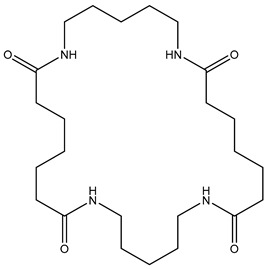
cyclic hexamer theoretical m/z: 679.512	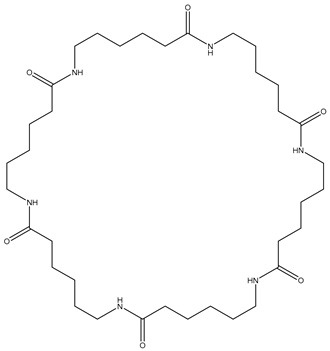	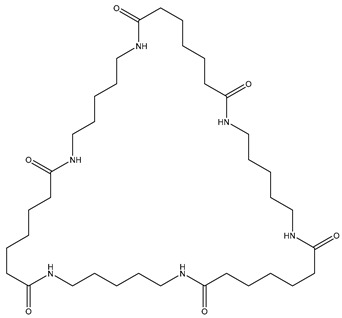

**Table 3:** Results of the IM-QTOF measurements for the different marker compounds of the polyamide grades.

m/z	Polyamide Grade	Retention Time/min	Drift Time/ms	^DT^CCS_N2_/Å^2^
227.1760 (cyclic dimer)	6	2.13	19.87	150.4
	6.6	2.13	20.83	157.8
340.260 (cyclic trimer)	6	2.34	23.93	178.8
453.3441 (cyclic tetramer)	6	2.56	28.35	209.6
	6.6	2.56	29.49	214.7
566.3890 (cyclic pentamer)	6	3.10	31.47	231.5
679.4339 (cyclic hexamer)	6	3.61	34.79	255.1
	6.6	3.33	35.67	261.6
198.1858	12	1.45	19.46	148.5
395.3638	12	1.41	27.23	202.0
592.5417	12	1.37	33.9	249.2

## Data Availability

The data presented in this study are available on request from the corresponding author.

## Supplementary Materials

The following are available online at https://www.mdpi.com/article/10.3390/polym13122032/s1, Table S1: Structures of the cyclic compounds of PA 12, Table S2: Structures of the cyclic compounds of PA 6, Table S3: Structures of the cyclic compounds of PA 6.6.
