# Supplementary material for: Differentiation of Polyamide 6, 6.6, and 12 Contaminations in Polyolefin-Recyclates Using HPLC Coupled to Drift-Tube Ion-Mobility Quadrupole Time-of-Flight Mass Spectrometry
Source: Polymers (Basel). 2021 Jun 21;13(12):2032. doi: 10.3390/polym13122032 (PMC8235147; doi:10.3390/polym13122032)
Supplement: Supplementary file 1 [file polymers-13-02032-s001.zip › polymers-1264322-supplementary.pdf]

Table S1. Structures of the cyclic compounds of PA 12.

Name	Chemical formula	theoretical m/z	chemical structure
laurolactam, Azacylcotridecane-2-one	$C_{12}H_{23}NO$	198.1858	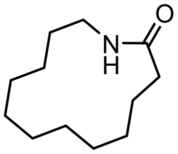
1,14-diazacyclohexacosane-2,15-dione	$C_{24}H_{46}N_2O_2$	395.3638	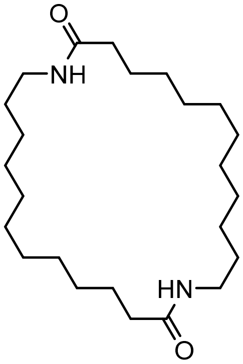
1,14,27-triazacyclononatriacontane-2,15,28-trione	$C_{36}H_{69}N_3O_3$	592.5417	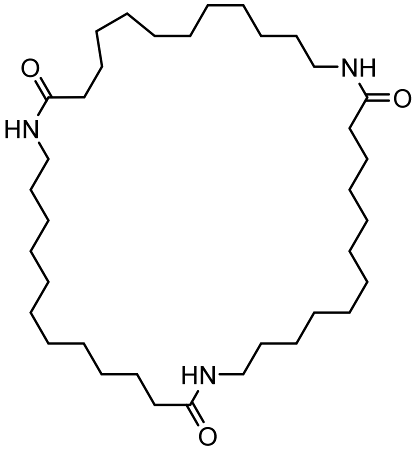

Table S2. Structures of the cyclic compounds of PA 6.

Name	Chemical formula	theoretical m/z	chemical structure
ε-caprolactam, Azepan-2-one	$C_6H_{11}NO$	114.0919	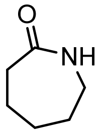
caprolactam cyclic dimer, 1,8-Diazacyclotetra-decane-2,9-dione	$C_{12}H_{22}N_2O_2$	227.1760	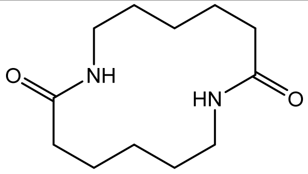
caprolactam cyclic trimer, 1,8,15-Triazacyclo-heneicosane-2,9,16-trione	$C_{18}H_{33}N_3O_3$	340.2600	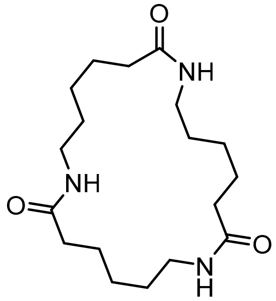
caprolactam cyclic tetramer, 1,8,15,22-Tetraazacyclo-octacosane-2,9,16,23-tetrone	$C_{24}H_{44}N_4O_4$	453.3441	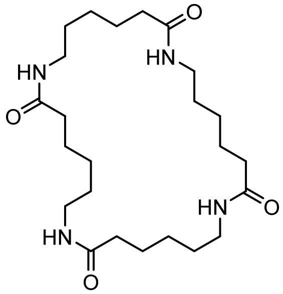
caprolactam cyclic pentamer, 1,8,15,22,29-Pentaazacyclopenta-triacontane-2,9,16,23,30-pentone	$C_{30}H_{55}N_5O_5$	566.3890	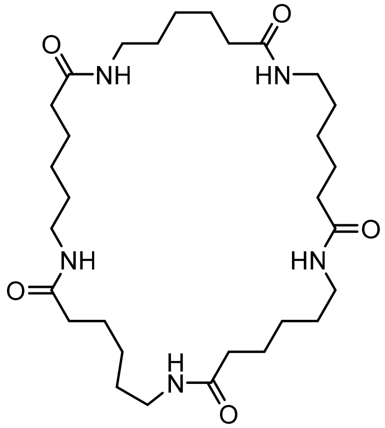

caprolactam cyclic hexamer, 1,8,15,22,29,36-<br><br>Hexaazacyclodotetracont<br><br>ane-2,9,16,23,30,37-hexone	$C_{36}H_{66}N_6O_6$	679.4339	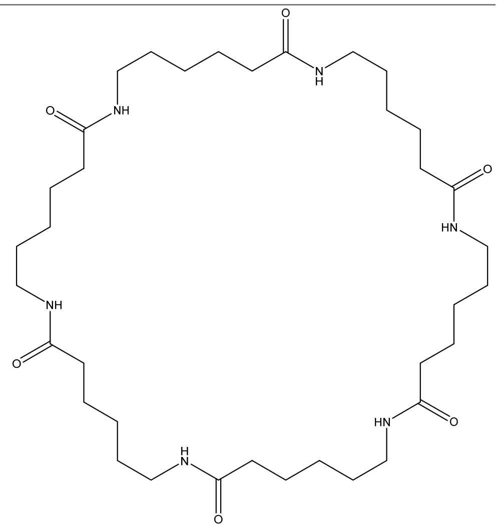

Table S3. Structures of the cyclic compounds of PA 6.6.

Name	Chemical formula	theoretical m/z	chemical structure
1,7-Diazacyclotetra-decane-8,14-dione	$C_{12}H_{22}N_2O_2$	227.1760	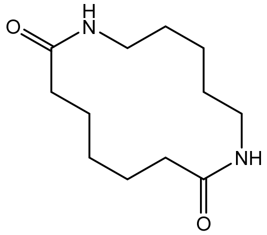
1,7,15,21-Tetraazacyclo-octacosane-8,14,22,28-tetrone	$C_{24}H_{44}N_4O_4$	453.3441	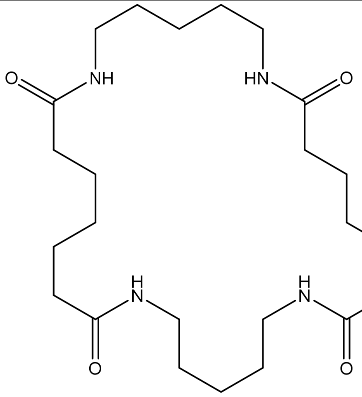
1,7,15,21,29,35-Hexaazacyclodotetracontane-8,14,22,28,36,42-hexone	$C_{36}H_{66}N_6O_6$	679.4339	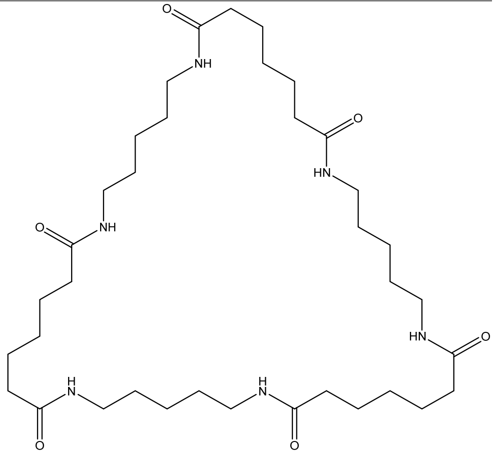
